# *Streptococcus intermedius*: An Unusual Case of Purulent Pericarditis

**DOI:** 10.1155/2017/5864694

**Published:** 2017-08-28

**Authors:** Kara J. Denby, Ryan D. Byrne, Oscar G. Gómez-Duarte

**Affiliations:** ^1^Departments of Internal Medicine and Pediatrics, Vanderbilt University Medical Center, Nashville, TN, USA; ^2^Division of Pediatric Infectious Diseases, University at Buffalo, The State University of New York, Buffalo, NY, USA

## Abstract

Purulent pericarditis is a rare diagnosis with life-threatening implications due to the rapid accumulation of pericardial material, swiftly progressing to tamponade physiology. The nature of its quickly evolving and severe implications demands a low threshold for diagnostic consideration where appropriate. We present an unusual case of purulent pericarditis secondary to* Streptococcus intermedius *in a previously healthy male adolescent without traditional risk factors, which raises the question of whether emergent* S. intermedius* species may have acquired novel molecular mechanisms.

## 1. Introduction

*Streptococcus anginosus *group (SAG), previously described as the* S. milleri* group, includes three bacterial species,* S. anginosus, S. intermedius, *and* S. constellatus*, all of them with a proclivity for abscess formation [1]. As a component of the microbiome, they are found in the oral cavity, gastrointestinal tract, and genitourinary tract. While they are associated with abbesses in brain, liver, abdomen, and lung, they have rarely been described as a cause of purulent pericarditis [1]. Prior mechanisms for development of purulent pericarditis include contiguous bacterial spread from an intrathoracic site, hematogenous spread, extension from a myocardial site, perforating injury or surgery, and extension from a subdiaphragmatic site [2]. Bacterial infections with SAG have been reported in children, though rarely as the cause of cardiac infections [1]. Within this bacterial group, only one case of purulent pericarditis due to* S. intermedius* in a child with local anatomic and histopathologic abnormalities has been previously reported [3]. Here we describe a case of purulent pericarditis secondary to* Streptococcus intermedius* in a previously healthy adolescent male with no host or environmental risk factors present.

## 2. Case Presentation

**Figure 1:**
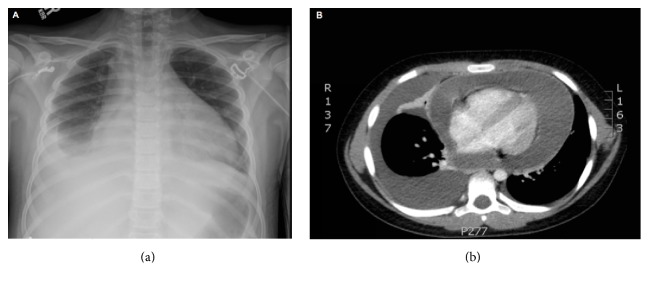
(a) Admission chest X-ray demonstrating enlarged cardiac silhouette with associated right-sided pleural effusion. (b) Computed tomography showing large circumferential pericardial effusion and associated pleural effusion.

A 12-year-old adolescent boy presented to the Monroe Carell Jr. Children's Hospital, Vanderbilt University Medical Center, Nashville, Tennessee, USA on July 3, 2015, with chief complaints of fever and epigastric abdominal pain that had been preceded by three weeks of intermittent fever and cough. On presentation, he was ill-appearing, afebrile (temperature of 36.6°C), tachycardic (heart rate of 138 beats/min), and tachypneic (respiratory rate of 40 breaths/min), with a blood pressure of 121/84 mmHg. Vital sign abnormalities were also notable for a 16 mmHg pulsus paradoxus. Cardiopulmonary physical exam revealed a pericardial friction rub without murmur or gallop, subcostal retractions, and diminished bibasilar breath sounds with otherwise clear lung fields. Abdominal exam revealed hepatomegaly, abdominal distension, and diffuse tenderness. Oral exam showed good dentition. Blood cell counts were notable for a leukocytosis with a white blood cell count of 31 × 10(3)/mcL, anemia with a hemoglobin of 9.7 gm/dL, severely elevated inflammatory markers (ESR 78 mm/hr, CRP 202.9 mg/L), moderately elevated transaminases (AST 939 units/L, ALT 351 units/L), coagulopathy (INR 3.3), and a lactic acidosis (lactate 6.9 mmol/L). An electrocardiogram was obtained, showing diffuse ST-segment elevations and PR-segment depressions. Chest X-ray (Figure 1) was notable for an enlarged cardiac silhouette and moderate right-sided pleural effusion, without evidence of pneumonia. Computed tomography of the chest was obtained showing a large circumferential pericardial effusion, a moderate right-sided pleural effusion, hepatomegaly, and moderate ascites. Echocardiography showed a large pericardial effusion, measuring 3.1 cm in the apical four-chamber view, with large clumping of echogenic material, and tamponade physiology demonstrated by right atrial and ventricular collapse with marked respiratory variation in mitral and aortic flows. An emergent subxiphoid pericardiocentesis was performed with drainage of 1,500 mL of grossly purulent yellow to green pericardial fluid. Gram's staining of the pericardial fluid showed Gram positive cocci in chains. He was initiated on empiric vancomycin, piperacillin-tazobactam, and doxycycline. Fluid culture grew alpha hemolytic streptococci that ultimately speciated as pansensitive* S. intermedius* using the Phoenix system (Becton Dickinson Diagnostic Systems, Sparks, MD). Histologic analysis of the pericardium was consistent with fibrinopurulent pericarditis. A thoracentesis was also performed draining 300 mL of serous pleural fluid. Culture of pleural fluid was negative. Despite the above interventions, our patient continued to have purulent drainage from the pericardiocentesis site and recurrent fevers. An abdominal ultrasound obtained to evaluate for intra-abdominal abscess was negative. Given the evidence for persistent infection, he was taken to the operating room for a pericardial window, washout, and pericardial drain placement with subsequent clinical improvement. The pericardial drain was removed on postoperative day 14. He was treated with a six-week course of ceftriaxone 2 grams/day with complete resolution of his symptoms.

## 3. Discussion

Historically purulent pericarditis was not an uncommon occurrence in pediatric patients, typically associated with pneumococcal pneumonia, and often fatal [4]. Purulent pericarditis is now rare in pediatric patients. Furthermore, purulent pericarditis secondary to SAG bacteria is exceedingly rare [5]. However, it is important to note that the SAG bacteria appear to have a predilection for causing more severe invasive infections, such as in infective endocarditis, when compared with other alpha hemolytic streptococci species [6]. This is a unique case of purulent pericarditis in a previously healthy adolescent patient with an undisturbed pericardial anatomy.

Purulent pericarditis is a rare diagnosis with life-threatening implications due to the rapid accumulation of pericardial material that may quickly progress to tamponade physiology. Our patient presented with complaints of fever and abdominal pain and was found to be acutely ill-appearing with tachycardia and tachypnea. Although rare, even in the absence of typical mechanisms by which purulent pericarditis often develops, pericardial disease, including purulent pericarditis, should be considered in the differential for all patients with symptoms similar to those listed above. The absence of more classic symptoms, such as positional chest pain and dyspnea, should not exclude the diagnosis since these are often not present. Interestingly, hepatomegaly, which our patient displayed on presentation, has been noted to be the most helpful clinical sign in pediatric patients, being present in 100% of patients under the age of 16 years with purulent pericarditis [4].

The mechanism used by* S. intermedius* to reach our patient's pericardium is unclear. It is possible that our patient's prodrome of fever and cough was associated with a viral pericarditis resulting in an inflammatory pericardial fluid collection prone to seeding with transient bacteremia from an oral or gastrointestinal source. Genome sequencing studies and a genome-wide comparative analysis have identified multiple putative virulence factors within the SAG group, including* S. intermedius* [7]. Adhesions, invasion proteins, spreading factors, cell wall proteins, and the* Streptococcus* invasion locus (Sil) two-component regulator of virulence present in* Streptococcus *spp. have also been found to be present in the SAG group [8, 9]. It is also possible that this particular* S. intermedius* isolate may have undergone a known* Streptococcus* genomic evolution pathway such as horizontal gene transfer as a mechanism of virulence factor acquisition resulting in an unusually hypervirulent* S. intermedius* strain [10].

## 4. Conclusions

In summary, this case report illustrates the ability of* S. intermedius *to cause a life-threatening purulent pericarditis in an otherwise healthy adolescent without typical risk factors and raises the question of whether emergent* S. intermedius* species have acquired novel molecular mechanisms of pathogenesis suitable for scientific exploration.

## Abbreviations

ALT: Alanine aminotransferase
AST: Aspartate aminotransferase
CRP: C-reactive protein
ESR: Erythrocyte sedimentation rate
INR: International normalized ratio
SAG: * Streptococcus anginosus* group.
